# Mosquito Meets Crystal

**DOI:** 10.1021/acs.accounts.6c00351

**Published:** 2026-08-30

**Authors:** Jingxiang Yang, Xiaolong Zhu, Bryan Erriah, Alexander G. Shtukenberg, Michael D. Ward, Bart Kahr

**Affiliations:** † State Key Laboratory of Elemento-Organic Chemistry, Frontiers Science Center for New Organic Matter, National Engineering Research Center of Pesticide, College of Chemistry, 12538 Nankai University, Tianjin 300071, People’s Republic of China; ‡ Pharmaceutical Analysis & Digital Technologies, 2793 Merck & Co., Inc., Rahway, New Jersey 07065, United States; § Department of Chemistry and Molecular Design Institute, 5894 New York University, 24 Waverly Place, New York, New York 10003-6688, United States

## Abstract

Combatting malaria, a disease afflicting more than 250 million people annually according to the World Health Organization (WHO), requires insecticides as part of integrated vector management. Indoor residual spraying (IRS) of crystalline contact insecticides and insecticide-treated bed nets (ITNs) decorated with insecticide crystals are estimated to have reduced malaria mortality in Africa by 60% in the 21st century. However, resistance now threatens malaria control, spurring the development of new insecticides, a process that requires substantial resources, anddiscovery time while posingenvironmental risks. Improving the effectiveness of compounds currently in use may be preferable. This *Account* provides a brief history of contact insecticides and describes discoveries from our laboratory that suggest paths to faster-acting contact insecticides based on the engineering of solid-state forms, either amorphous or crystalline polymorphs, thereby potentially obviating the need for new chemicals. Contact insecticides are thought to affect insects by absorption through the footpads. Prior to our 2017 report on the structure of a second solid form of DDT, crystal polymorphism was not optimized for contact insecticides. Comparative analysis of knockdown times for flies and mosquitoes against polymorphs of well-known crystalline contact insecticides including DDT and its analogs, as well as lindane, deltamethrin, and imidacloprid, established a link between thermodynamic crystal stability and insect knockdown speed. The relationship between the knockdown by a crystalline contact insecticide and its crystal structure ultimately arises from crystal thermochemistry. Weak intermolecular interactions in complex systems and associated shallow potential energy hypersurfaces readily lead to crystalline polymorphs with different molecular organizations that vary in crystal free energies and associated bioavailabilities. Notably, a new form of deltamethrin was 12 times more active than the commercial form, and the least stable polymorph of imidacloprid was six and nine times more active against susceptible *Anopheles* and *Aedes* mosquitoes, respectively, than the commercial, thermodynamically stable form. Some of these metastable polymorphs were found to be stable against transformation to their thermodynamically stable forms for months in an idealized laboratory setting, approaching WHO guidelines for practical use. The observation of differing polymorph effectiveness demonstrates that tarsal absorption of molecules from the crystal surfaces by insects is a key step, and likely a limiting step, in the insecticidal action. Indeed, a persistently amorphous form of deltamethrin dispersed on chalk exhibited dramatically increased efficacy against deltamethrin-resistant *Anopheles* mosquitoes from Burkina Faso, revealing that an increased rate of insecticide uptake overwhelmed all the resistance mechanisms tested. Collectively, this work argues that manipulation of the solid-state chemistry of contact insecticides is a viable strategy for mitigating insect-borne diseases and one that should be considered along with others in integrated vector management.

## Graphical abstract

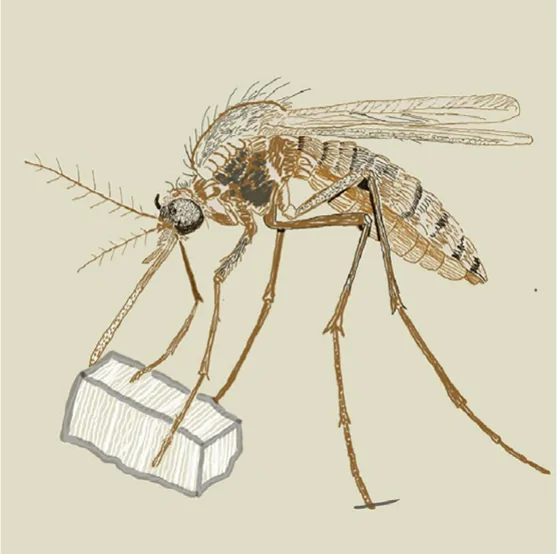

## Key References

- Yang, J.; Hu, C. T.; Zhu, X.; Zhu, Q.; Ward, M. D.; Kahr, B. DDT polymorphism and the lethality of crystal forms. *Angew. Chem.* **2017**, *129*, 10299–10303.^1^
- Yang, J.; Erriah, B.; Hu, C. T.; Reiter, E.; Zhu, X.; López-Mejías, V.; Carmona-Sepúlveda, I. P.; Ward, M. D.; Kahr, B. A deltamethrin crystal polymorph for more effective malaria control. *Proc. Nat. Acad. Sci. U.S.A.* **2020**, *117*, 26633–26638.^2^
- Zhu, X.; Hu, C. T.; Erriah, B.; Vogt-Maranto, L.; Yang, J.; Yang, Y.; Qiu, M.; Fellah, N.; Tuckerman, M. E.; Ward, M. D.; Kahr, B. Imidacloprid crystal polymorphs for disease vector control and pollinator protection. *J. Am. Chem. Soc.* **2021**, *143*, 17144–17152.^3^
- Carson, J.; Erriah, B.; Herodotou, S.; Shtukenberg, A.; Smith, L.; Ryazanskaya, S.; Ward, M. D.; Kahr, B.; Lees, R. S. Overcoming Insecticide resistance in *Anopheles* mosquitoes by using faster-acting solid forms of deltamethrin. *Malaria J.* **2023**, *22*, 129.^4^

## 1 Introduction

The World Health Organization’s Global Malaria Eradication Programme of 1955–1969 was insufficient to solve a widespread human health challenge, despite international cooperation.^5^ In hindsight, the failure to reduce malaria transmission to zero was a consequence of the narrow focus on two chemical interventions, the insecticide DDT (1,1,1-trichloro-2,2-bis­(4-chlorophenyl)­ethane, Scheme 1), which became notorious for its ecological impact^6,7^ —two million tons of DDT were slathered on the planet—and the antimalarial drug chloroquine. Both were overused, resulting in the development of resistance in the disease-transmitting *Anopheles* mosquitoes and in the *Plasmodium* parasite, respectively. This has prompted new innovations for the control of malaria and other vector-borne infectious diseases, whether through new insecticide compounds or still-evolving biological solutions, including vaccines and gene drives.^8^ New insecticides, however, are continually challenged by the development of resistance and environmental concerns. Biological interventions are yet imperfect and risky. Whatever best strategies emerge, their success relies on international collaboration, which, unfortunately, is ebbing at the time of this writing.

**Scheme 1:**
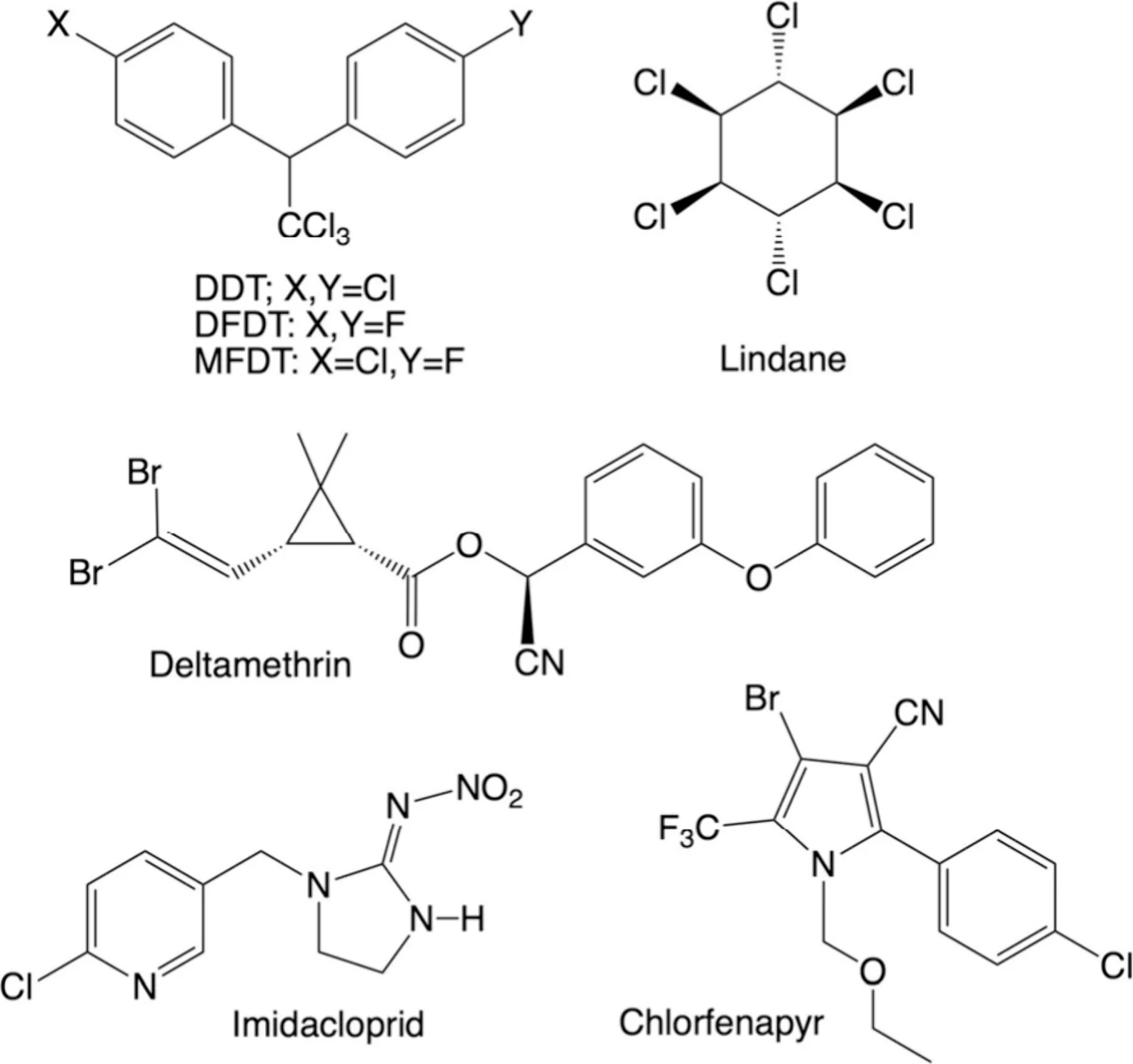
Molecular Structures of the Insecticides Discussed in This Article

The collaboration among the authors of this *Account* began when DDT entered our laboratory to address two questions involving crystal growth mechanisms^9–11^ unrelated to its insecticidal properties. During this research, we characterized a second crystal polymorph of DDT^12,13^ and hypothesized a possible connection between polymorphism and insecticidal activity. DDT is a contact insecticide; it works when insects land on a crystal surface and detach molecules that enter the organism (Figure 1).

**Figure 1:**
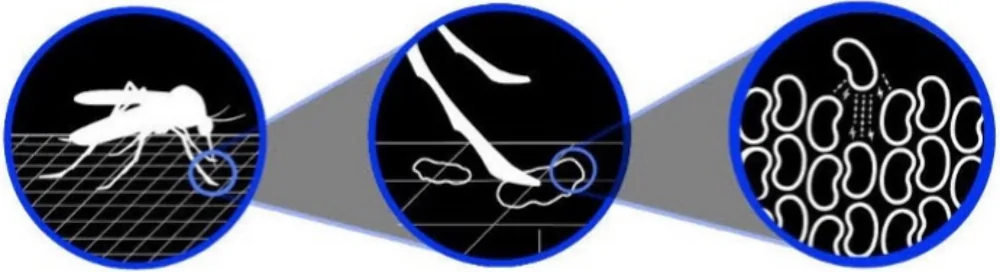
Schematic interaction of a mosquito and the surface of a crystalline contact insecticide, zooming in from the left panel to the right. Upon contact of the insect footpad with the crystal surface, molecules must detach and subsequently be absorbed by the footpad, among other potential points of entry. Schematic provided courtesy of Maria Constanza Ferreira.

Crystalline contact insecticides function only after their molecules have completed a long journey. When a mosquito walks on a hydrophobic crystalline insecticide it presumably picks up molecules or small particles that dissolve in the hydrocarbon-rich epicuticle, the outermost part of the insect’s tarsi (i.e. feet), among other points of entry. Diffusion then carries the molecules into the exoskeleton, the procuticle, which contains proteins and chitin.^14^ Ultimately, the molecules enter the hemolymph, the internal fluids of a mosquito, and then compromise an active biochemical site. Here, we are focused on the points of departure in these journeys, where differences in the absorption of molecules from crystal surfaces by the insect play a role.

Crystal–whole organism interactions remain crucial to efforts to control malaria today, with contact insecticides that have replaced DDT, so-called synthetic pyrethroids.^15^ Pyrethroid crystals cover the surfaces of some three billion insecticide-treated nets (ITNs), the foremost intervention in contemporary malaria control in the 21st century.^16^ Alternatively, homes are routinely protected against invading mosquitoes by indoor residual spraying (IRS) of contact insecticide formulations on the inside walls of homes.^17^ Indeed, for almost a century, molecular crystals have been involved wherever humans have attempted to control deadly mosquitoes. Our experience prompted a simple question: “Which crystals”? This *Account* focuses on our efforts to address the extent to which the solid states of polymorphous and amorphous insecticides can affect bioactivity and thereby impact in the control of infectious diseases.

## 2 Knockdown Rule

The isolation and characterization of the second DDT polymorph (Form II),^1^ which has a crystal structure distinct from the known Form I (Figure 2A,B), presented a murder mystery of a kind: If two forms are possible, which is the more effective culprit? We found that the metastable Form II was more active against *Drosophila melanogaster* (fruit flies), excellent proxies for mosquitoes,^18^ in contact with powdered crystals as measured by median knockdown times (denoted as KD_50_, the time required for 50% of the insects to become immobile). Smaller KD_50_ values for Form II suggested a simple hypothesis supported by chemical intuition that we aspired to generalize: The higher the free energy of the solid form of a contact insecticide, the greater its effectiveness. The most stable polymorph, likely the dominant form in commercial insecticide formulations, has the lowest free energy and should be the *least* active against insects because it would surrender its molecules to the insect tarsi (i.e., feet) less efficiently. Conversely, polymorphs with higher free energies should be the faster acting, and amorphous forms faster acting still.

**Figure 2:**
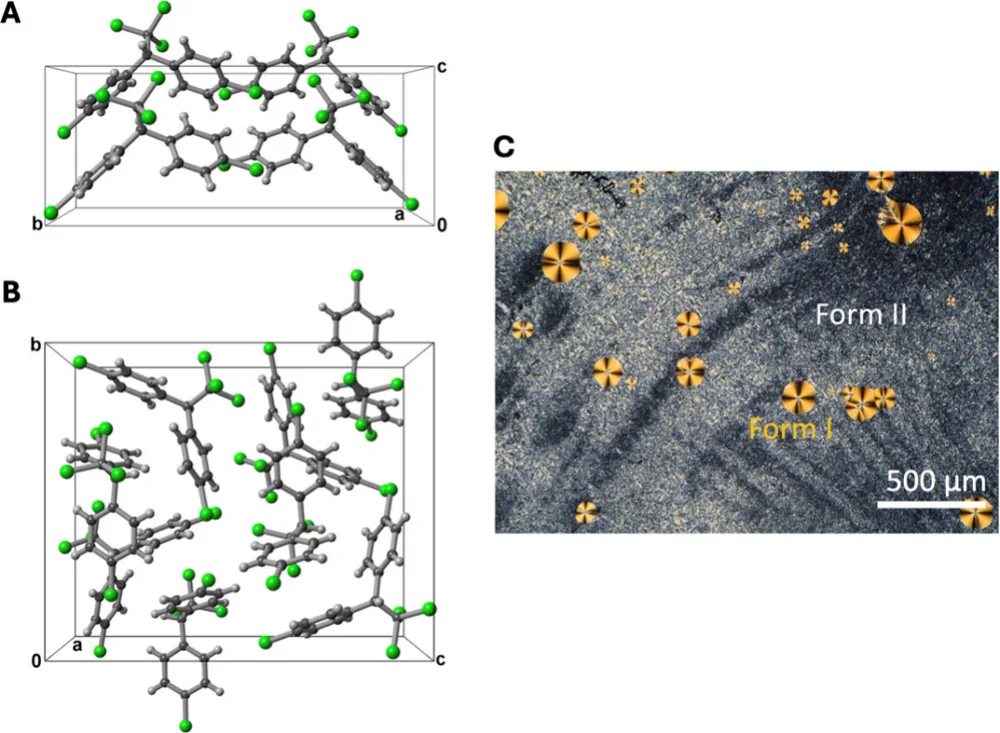
Crystal structures of (A) DDT Form I and (B) DDT Form II. The structure of Form I, previously available in Cambridge Structural Database (Refcode CPTCET10) was redetermined. Atom colors: hydrogen, white; carbon, gray; chlorine, green. (C) Crystallization of DDT Form I (spherulites with extinction crosses) and Form II (chaotic texture), from the melt at 0 °C. Adapted with permission from ref 1, copyright 2017, John Wiley and Sons.

The hypothesis correlating thermodynamic stability and bioavailability was subsequently tested for other insecticides and found to hold generally (Table 1). The observation that polymorphs have distinctly different knockdown speeds must be a consequence of different rates of uptake from solid surfaces. Once inside the organism, the origin of the molecules is irrelevant. Naturally, there are strong correlations with the achievements of the pharmaceutical industry related to the bioavailability of solid-state formulations.^19^ In controlling arthropods with crystals, however, the active ingredient begins outside the organism as opposed to being ingested and delivered within.

**Table 1:** Correlation between Free Energy and Knockdown Rankings of Contact Insecticides

Insecticide^a^	Organism tested	Free energy^b^	KD_50_ ^b^	Reference
DDT	*Drosophila melanogaster*	II > I	II > I	^1^
DDT	*Anopheles quadrimaculatus*, *Aedes aegypti*	*a* > I	*a* > I	^1^,^17^
DFDT	*Drosophila melanogaster*, *Anopheles quadrimaculatus*, *Aedes aegypti*	*a* > II > I	*a* > II > I	^20^
(*RS*)-MFDT	*Drosophila melanogaster*	*a* > III > II > I	*a* > III > II > I	^17^
(*RS*)-MFDT	*Anopheles quadrimaculatus*, *Aedes aegypti*	*a* > I	*a* > I	^17^
(*R*)-MFDT	*Drosophila melanogaster*	*a* > I	*a* > I	^17^
(*S*)-MFDT	*Drosophila melanogaster*	*a* > I	*a* > I	^17^
Lindane	*Drosophila melanogaster*	III > II > I	III > II > I	^21^
Deltamethrin	*Drosophila melanogaster*, *Anopheles quadrimaculatus*, *Aedes aegypti*	II > I	II > I	^2^
Imidacloprid	*Drosophila melanogaster*, *Anopheles quadrimaculatus*, *Aedes aegypti*, *Culex quinquefasciatus*	IX > VI > IV > I	IX > VI > IV > I	^3^
Chlorfenapyr	*Drosophila melanogaster*	IV > III ≈ II > I	IV ≥ III ≈ II ≈ I	^22^
Chlorfenapyr	*Anopheles quadrimaculatus*	II > I	II ≈ I	^19^

a Molecular formulas are shown in Scheme 1.

b Roman numbers correspond to different polymorphs; *a* is amorphous solid.

If distinct polymorphs liberate molecules at different rates – a Hammond postulate-based argument – symmetry-independent faces of a single crystal should likewise liberate molecules at different rates, too. Indeed, when fruit flies were exposed to different facets of a large crystal of DDT Form I (2 cm in the longest direction), the KD_50_ values decreased in the order (001) ≈ (001̅) > (1̅20) > (1̅10) (Figure 3).^23^ This trend correlated well with attachment energies (roughly proportional to surface energies) calculated for DDT, thereby corroborating the knockdown rule.

**Figure 3:**
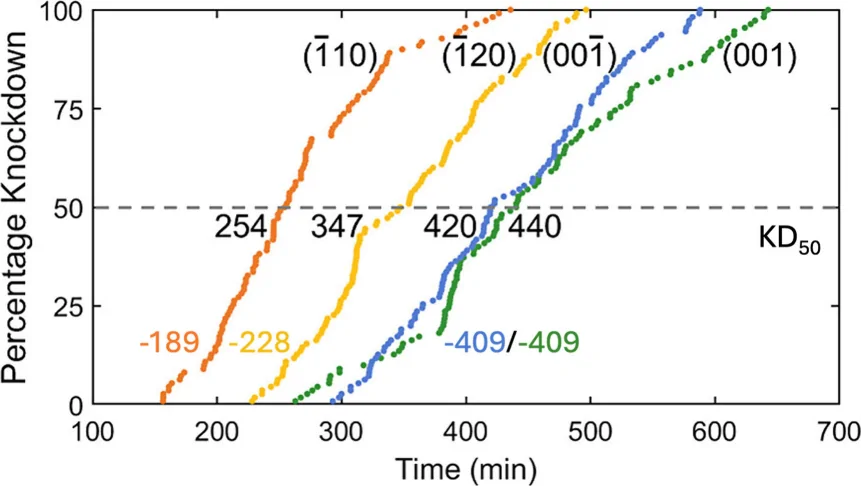
Knockdown times for *Drosophila melanogaster* exposed to individual faces of DDT Form I single crystals. Each symbol corresponds to one female. The dispersion of points for any one face is due to biological variability of individual organisms. The median knockdown time for each curve is denoted by its intersection with the horizontal KD_50_ marker. The color-coded numbers at the bottom of the figure represent the computed attachment energies in kJ/mol. Adapted with permission from ref 23, copyright 2024 American Chemical Society.

This work speaks to the mechanism of contact insecticide activity. Symmetry-independent facets must have unique surface energies. Molecules with higher chemical potentials on some surfaces, or on surfaces of metastable polymorphic forms, would be expected to surrender molecules to the epicuticle more rapidly. Nevertheless, the individual chemical steps in the transport process are not well understood.

## 3 Early Years of Synthetic Insecticides

Following our serendipitous encounter with DDT, we dug into the earliest literature from the era of synthetic insecticides, beginning with the discovery of the insecticidal activity of DDT by Müller at Geigy in Switzerland. Müller was later awarded the Nobel Prize based on the premature presumption that insect-borne infectious diseases would be eradicated.^24^ Surprisingly, a faster-acting difluoro congener, 4,4′-(2,2,2-trichloroethane-1,1-diyl)­bis­(fluorobenzene) (DFDT, Scheme 1)^25^ was developed in Germany during World War II. Forty tons per month were synthesized by Höchst.^26^ In his Nobel address, Müller indicated that DDT was effective but slow-acting. He suspected that speed mitigates the development of resistance and advocated for DFDT.^27^ Despite its alleged performance, it vanished from public health considerations, not only because of the ruin of German industry but also because DFDT was dismissed by Allied inspectors in 1945: “The German claims as to the superior insecticidal action of their [DFDT], in comparison to DDT, are clearly not supported by their meager and inadequate tests against houseflies.”^28^ Other assessments were pejorative. Consequently, DDT became the dominant postwar solution to the mitigation of vector-borne disease, including malaria. A vast infrastructure for its synthesis was already established in the United States. DFDT was never considered for the Malaria Eradication Programme, to the best of our knowledge.

As an exercise in the experimental history of science, we evaluated DFDT solid forms against *Drosophila* melanogaster as well as *Anopheles quadrimaculatus* and *Aedes aegypti* mosquitoes;^17^ the former genus includes the disease vectors for malaria while the latter species transmit Zika, yellow fever, dengue, and chikungunya, among other infectious diseases. These fluorous derivatives were faster acting than amorphous DDT (*a*-DDT), which was more active than its two crystalline forms (Figure 4). Moreover, dimorphous crystalline DFDT and a trimorphous monofluoro racemic congener (*RS*)-MFDT (1-chloro-4-(2,2,2-trichloro-1-(4-fluorophenyl)­ethyl)­benzene, Scheme 1) followed the knockdown rule; activity was correlated with thermodynamic instability.

**Figure 4:**
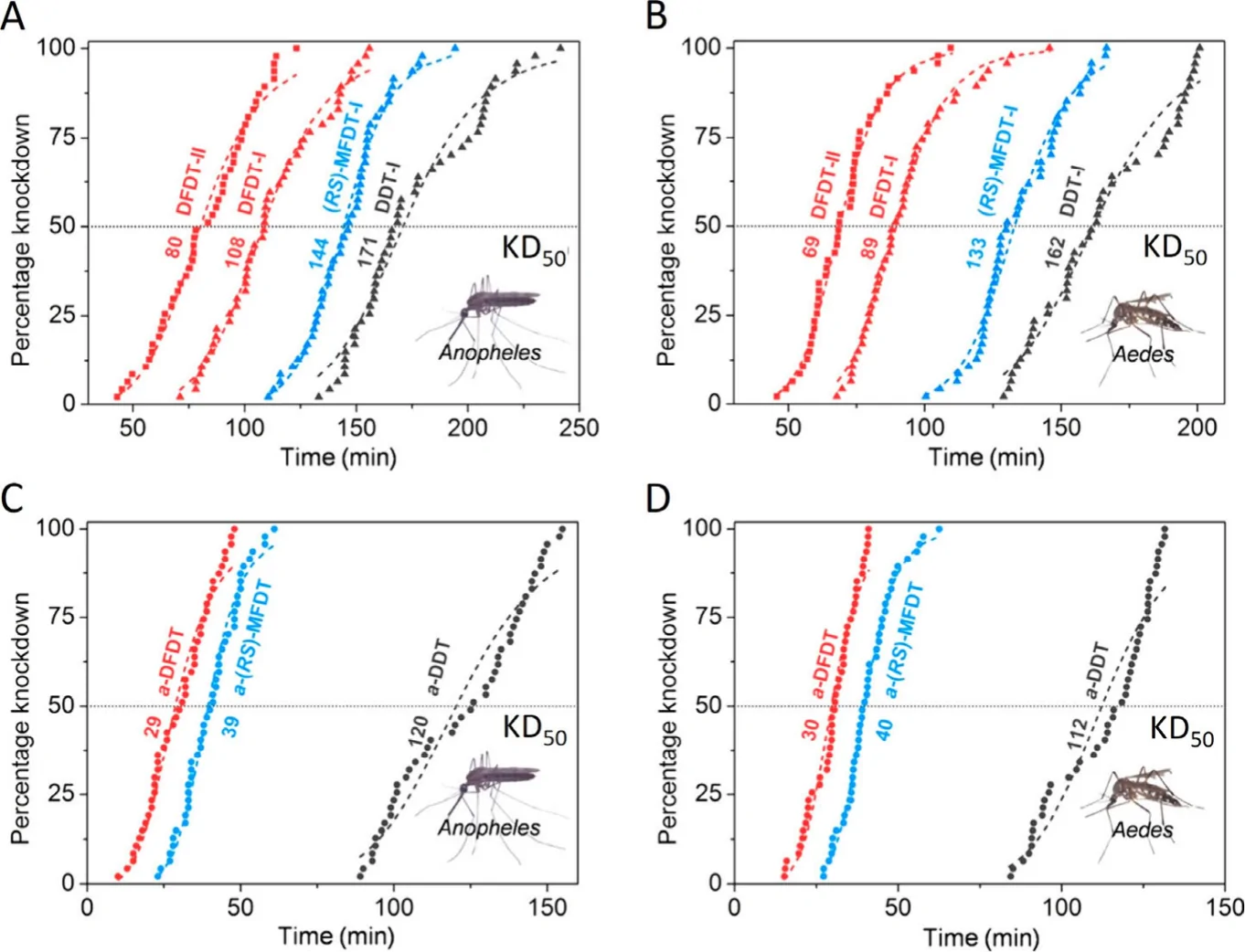
(A, B) Knockdown of crystalline forms of DFDT, (*RS*)-MFDT, and DDT for *Anopheles quadrimaculatus* (A) and *Aedes aegypti* (B). (C, D) Knockdown of amorphous forms *a*-DFDT, *a*-(*RS*)-MFDT, and *a*-DDT for *Anopheles quadrimaculatus* (C), *Aedes aegypti* (D). Dashed lines indicate logistic regression of knockdown-time curves. Adapted with permission from ref 20, copyright 2019 American Chemical Society.

Is it possible to envision a counterfactual history for the Malaria Eradication Programme of the mid-20th century? Would faster-acting DFDT have further reduced malaria transmission globally had it been used in lieu of DDT? No such comparison of DDT and DFDT was made when it might have been consequential, presumably because DDT was doing everything asked of it. As DDT and DFDT were eventually shown to be cross-resistant, this analysis cannot be performed in hindsight.^29^ Likewise, we cannot know about the comparative environmental consequences of the two compounds. History cannot be rerun.

The knockdown rule also applies to three polymorphs of lindane (1*R*,2*r*,3*S*,4*R*,5*r*,6*S*-hexachlorocyclohexane or γ-hexachlorocyclohexane, Scheme 1), a reviled persistent organic pollutant.^30^ One crystal structure of lindane was known^31,32^ before our laboratory described two more.^18^ The insecticidal activity, as measured by KD_50_, was correlated inversely with the relative stability of powdered crystals when evaluated against fruit flies. Average knockdown times were 42, 82, and 127 min, in order of the least stable crystal followed by most stable, respectively. For each ton of lindane manufactured between 1950 and 2009, 8–12 tons of noninsecticidal hexachlorocyclohexane stereoisomers were produced concomitantly, which are much more toxic to mammals. More than five million tons of such waste stereoisomers are heaped around the world.^27^ If the knockdown rule had been recognized in the last century, and the fastest-acting Form III was manufactured at the outset, perhaps the environmental waste burden would be reduced by two-thirds – still a disaster, but a credible story with wisdom for the future management of environmental toxins. As described in the next section, many insecticides for mitigating infectious diseases, whether currently approved for use or obsolete, can have dangerous consequences for nontarget organisms. The lesson of lindane is that the less manufactured the safer the environment will be overall.

## 4 Contemporary Contact Insecticides

Chlorinated insecticides such as DDT and lindane are no longer used because of their harm to the environment. The principal compounds currently used for controlling *Anopheles* mosquitoes are the synthetic pyrethroids. Foremost in this class is deltamethrin ((*S*)-cyano­(3-phenoxyphenyl)­methyl (1*R*,3*R*)-3-(2,2-dibromovinyl)-2,2-dimethylcyclopropane-1-carboxylate, Scheme 1). In 2020, only one solid form of deltamethrin had been described, Form I.^33^ We discovered that cooling a melt of Form I (mp = 100 °C) generated a metastable polymorph denoted Form II (Figure 5B), which grew as spherulites that were easily distinguishable from the more chaotic morphology of Form I (Figure 5A). The metastable Form II was surprisingly persistent at room temperature. When Forms I and II crystals were evaluated against susceptible North American *Anopheles quadrimaculatus* mosquitoes, the Form II knockdown time was seven times shorter (Figure 6), once again substantiating the knockdown rule – the higher energy solid form was faster acting. On heating deltamethrin dispersed on chalk past the melting point of Form I, the acceleration in knockdown times was 12-fold. Using a previously reported malaria transmission model^34^ and presuming a 12× faster-acting Form II was equivalent to a 12× increase in applied dose, the transmission of malaria and the development of resistance were predicted to decline substantially (Figure 7).

**Figure 5:**
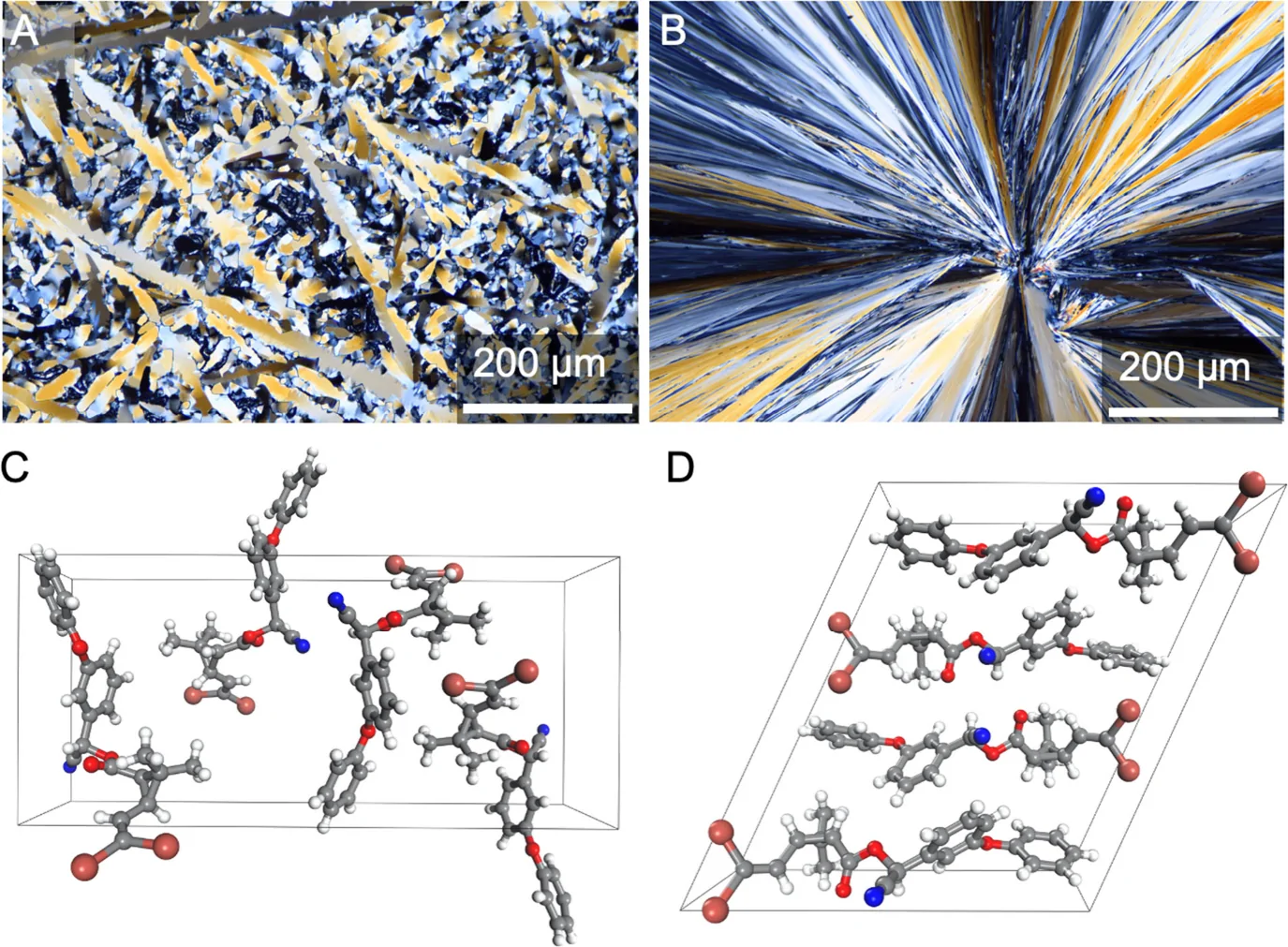
Melt-grown deltamethrin (A) Form I and (B) Form II observed between crossed polarizers. Single-crystal structures of (C) Form I and (D) Form II. Atom colors: hydrogen, white; carbon, gray; oxygen, red; nitrogen, blue. Adapted with permission from ref 2, copyright 2020 National Academy of Sciences.

**Figure 6:**
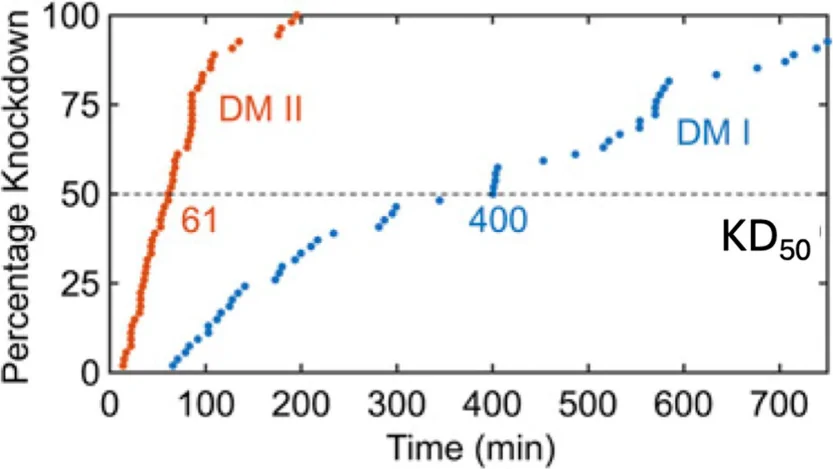
(Left) KDs for *D. melanogaster* exposed to crystalline films. DM I is deltamethrin Form I; DM II is deltamethrin Form II. Adapted with permission from ref 2, copyright 2020 National Academy of Sciences.

**Figure 7:**
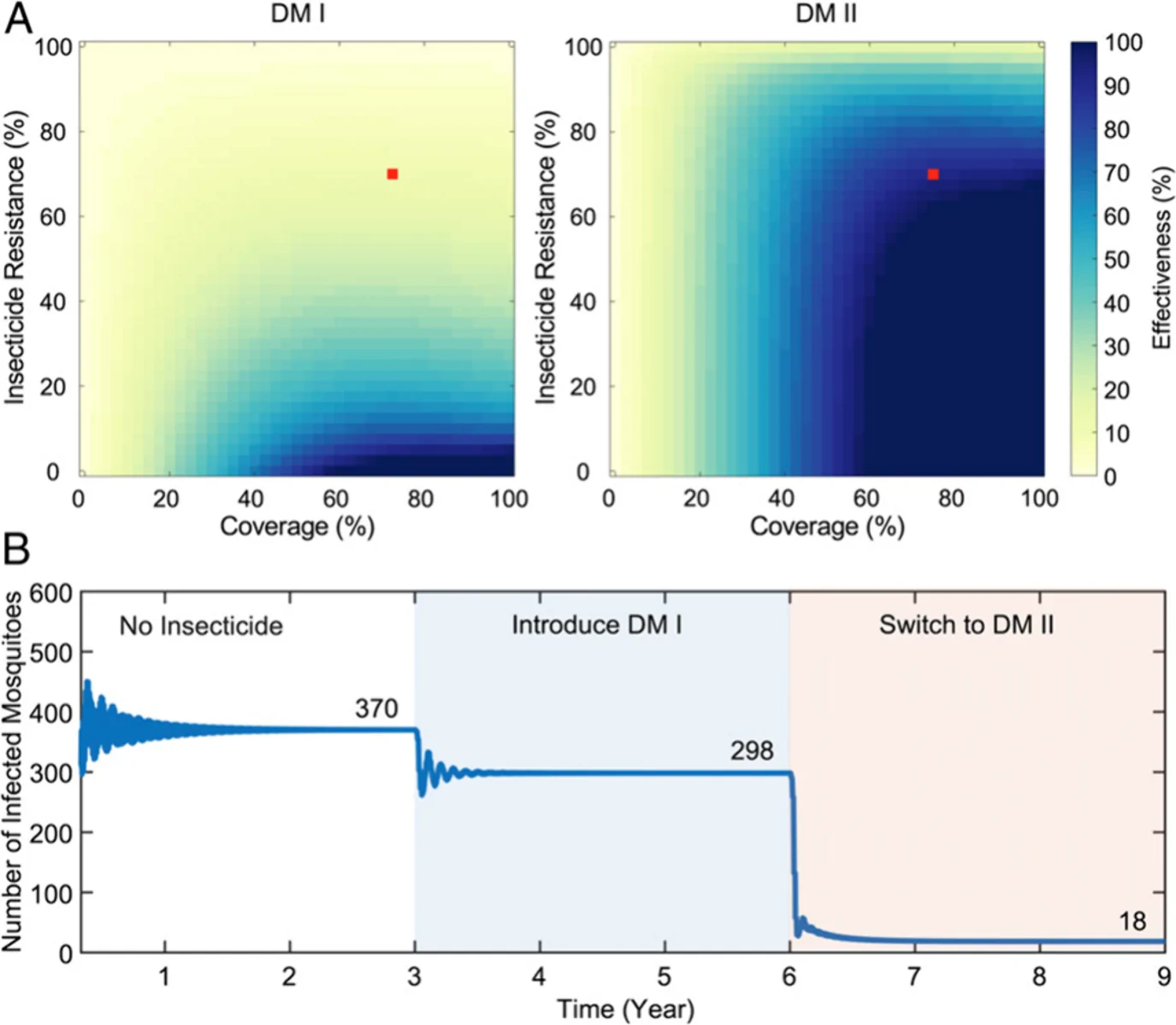
Simulation of malaria transmission dynamics according to a model in ref 34. (A) Effectiveness of IRS for inhibition of malaria transmission, using the same doses of deltamethrin Form I (Left, DM I) or Form II (Right, DM II) at varying levels of coverage and insecticide resistance with an initial human infection prevalence of 45%. Insecticide resistance corresponds to the percentage of mosquitoes that survive after contacting the applied dose of Form I. Coverage corresponds to the probability of a mosquito contacting the insecticide during a single resting episode. (B) Predicted reduction in population of infected mosquitoes upon application of deltamethrin Form I and then switching to Form II (coverage = 75%; resistance = 70%; infection prevalence = 45%, denoted in Figure 7A as red squares). The percentage of infected humans also dropped precipitously. Adapted with permission from ref 2, copyright 2020 National Academy of Sciences.

It was subsequently discovered that amorphous deltamethrin, not Form II, was generated from Form I in chalk by heating a gardening product (D-Fense Dust, 0.05 wt % deltamethrin on calcite) as purchased in a microwave oven.^35^ Tarsal contact bioassays subsequently conducted for resistant *Anopheles* species from Burkina Faso^4^ revealed that the heated D-Fense Dust with amorphous deltamethrin was substantially more effective with respect to knockdown and 24-h mortality, an accepted WHO metric, against mosquito strains that expressed a variety of resistance mechanisms (Figure 8). The 100% mortality can be explained by increased bioavailability. Enhanced release of the insecticide molecules from the higher free-energy solid forms presumably results in a higher concentration of insecticide in the organism, which in turn can overwhelm different molecular biochemical resistance mechanisms (e.g. target site resistance or metabolic resistance).

**Figure 8:**
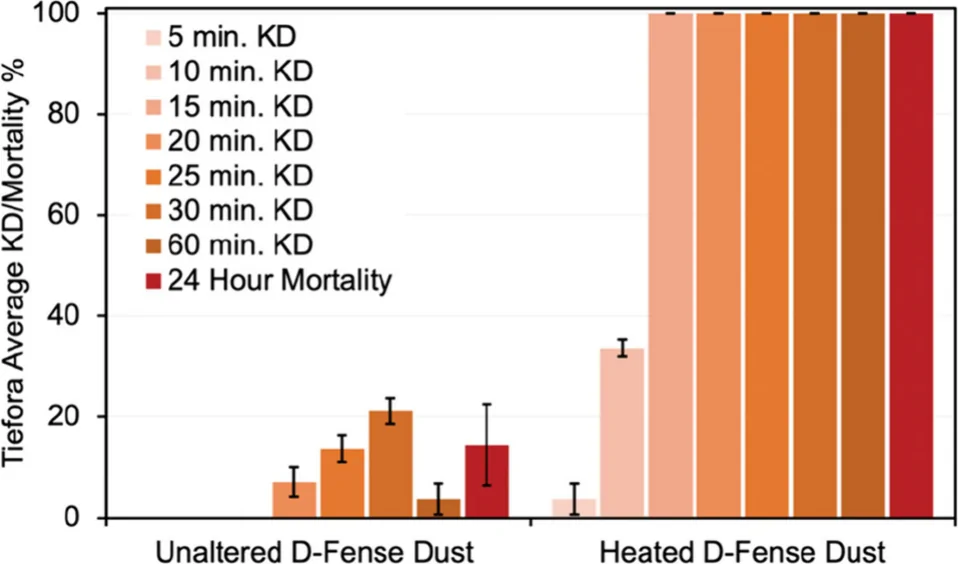
WHO tarsal assay. Average knockdown % and 24-hour mortality of pyrethroid-resistant *Anopheles arabiensis* Tiefora strain mosquitoes exposed to D-Fense Dust that was heated at 150 °C for 30 min and allowed to cool to room temperature. Error bars correspond to one standard deviation based on triplicate measurements. All resistant strains showed similar responses. Adapted with permission from ref 4, copyright 2023 Springer Nature, license http://creativecommons.org/licenses/by/4.0/.

Imidacloprid ((*E*)-*N*-(1-((6-chloropyridin-3-yl)­methyl)­imidazolidin-2-ylidene)­nitramide, Scheme 1), classified as a neonicotinoid, is currently one of the world’s most-used^36^ insecticides. Overuse poses serious threats to pollinators.^37^ Nevertheless, a neonicotinoid, clothianidin, was approved recently for controlling infectious disease vectors in indoor residual sprays.^38^ Human health and environmental health priorities are often in conflict, yet reducing neonicotinoid use is paramount. We described seven new imidacloprid polymorphs, adding to two known forms (Figure 9). The knockdown rule was corroborated once again; metastable crystal forms were as much as nine times faster acting than the commercial form against susceptible *Aedes*, *Anopheles*, and *Culex* mosquitoes. These results suggest that replacement of commercially available imidacloprid crystals (Form I) in agricultural space-spraying with any one of three new metastable polymorphs, Forms IV, VI, and/or IX, would reduce environmental exposure. We stress that for contact insecticides with life-saving public health functions and deleterious consequences for nontarget organisms, minimizing manufacture and exposure by controlling the activity in the solid state is a strategy that can be considered.

**Figure 9:**
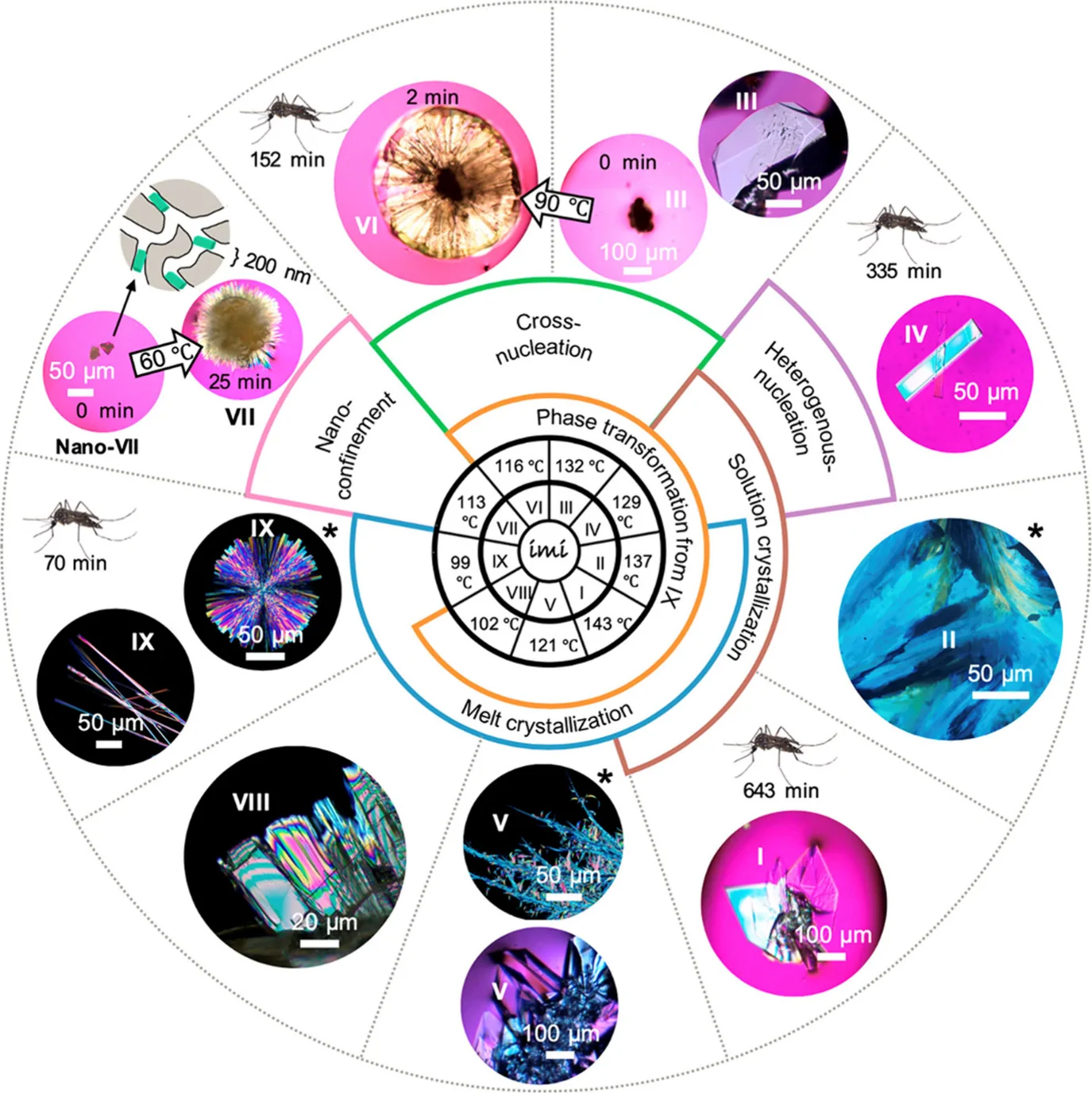
Imidacloprid polymorphism. The melting points and crystallization methods are labeled next to each polymorph. Polarized-light optical micrographs of samples confined between glass slides are labeled with *, which include spherulites of Form IX, chaotic textures of Form V, and flat crystalline domains of Form II grown from the melt at ambient temperature. Single crystals of Forms I, III, IV, and V grown from melt in the presence of respective crystal seeds were prepared on a microscope hot stage at 135, 130, 126, and 120 °C, respectively. Form VI was obtained through cross-nucleation on Form III in the melt at 90 °C. Form VII was obtained by seeding Form VII confined in controlled-pore (200 nm) glass into the melt at 60 °C. Single crystals of Forms VIII and IX grown from melt at 85 and 70 °C, respectively. Polymorphs accompanied by Aedes mosquito icons were selected for knockdown measurements. The median KD_50_s for female *Aedes* are given in minutes. Adapted with permission from ref 3, copyright 2021 American Chemical Society.

We also investigated the pro-insecticide chlorfenapyr (4-bromo-2-(4-chlorophenyl)-1-(ethoxymethyl)-5-(trifluoromethyl)-1*H*-pyrrole-3-carbonitrile, Scheme 1), a newly approved WHO active ingredient,^39^ which we found crystallized as four polymorphs.^19^ A pro-insecticide is an agent that is metabolized to the active form once inside an organism; with respect to chlorfenapyr, its ether appendage must be hydrolyzed enzymatically to generate the active compound. Forms I, II and III of chlorfenapyr are polytypes having similar crystal structures, suggesting similar free energies. Consequently, no significant difference in knockdown would be anticipated if uptake was the rate-determining step. Form IV has a distinctly different structure and it shows a greater activity (Table 1).

## 5 Outlook

Insecticide resistance is a global challenge. If we could map out how to practically solve this problem, we would be in possession of precious information. Our goals in this *Account* are more modest. We hope to alert epidemiologists and entomologists to the fact that synthetic contact insecticides that have been used routinely for >80 years involve the interactions of a whole organism with a crystalline solid. As such, determining which solid is consequential is imperative. Then we need the engagement of public health researchers to carry this idea to practical application, a matter that has become especially urgent since the disengagement of the United States from the World Health Organization and the degradation of the United States Agency for International Development.

The knockdown rule, which holds for all contact insecticides studied by our group, opens avenues for formulating more effective forms of insecticides to combat resistance and reduce environmental impact. The only caveat is the pro-insecticide chlorfenapyr, whose polymorphs expressed only small differences in solid-state structures and energies. Metabolic processes needed to activate the pro-insecticide could be rate-determining. The rule is now finding applications in other laboratories^40–42^ and even in industry.^43^ The observed correlation between thermodynamic free energy and knockdown times is consistent with chemical intuition, but the mechanistic details and interactions between whole organisms and crystals remain unclear (Figure 1). Nonetheless, the different efficacies of polymorphs, or different crystal surfaces of the same compound, strongly support detachment of molecules from the crystal as a key step required for transport through the tarsi. The differences in solid-form activities can only come from the crystal detachment step about which we need further collaborative assays. Rate measurements are essential for mechanistic insights into any chemical process. A common method for tracking insecticide molecule transport in organisms is autoradiography with ^14^C-labeled active ingredients.^44^ This, or some other method of quantitative imaging of dissected mosquitoes, will be required to confirm the implications of our observations. Mass spectrometry imaging should be useful for mapping dibromides like deltamethrin.^45^

Some of these highly active metastable solid forms described above did not transform to their less active thermodynamically stable forms for months in an idealized laboratory setting (e.g., heated deltamethrin dusts retained their activity for at least 19 months) approaching WHO guidelines for practical use. This needs to be tested in the field, however. In the battle against malaria, crystalline contact insecticides are most frequently used in ITNs. ITNs are widely regarded as the most successful malaria intervention since the Gates Foundation and the WHO reembraced the concept of malaria eradication in recent decades. Therefore, we have been shifting our focus toward crystallization in extruded polyethylene (PE) fibers – easily mimicked in the laboratory and evaluated by Raman and electron microscopies – that make up about half of the 3 billion ITNs distributed worldwide in the 21st century. PE nets supersaturated with synthetic pyrethroids abundantly produce abundant blooms ofcrystals on the surfaces, yet the identities of these crystals have not been reported. We have found that extruded PE fibers containing 1 wt % of the highly polymorphic noninsecticidal compound ROY (5-methyl-2-[(2-nitrophenyl)­amino]­thiophene-3-carbonitrile) exhibit 6 of the 14 known polymorphs on their surfaces (Figure 10A).^46^ Moreover, the fractions of different polymorphs evolved over time. Deltamethrin emerges from PE as droplets that then grow Form I crystals (Figure 10B). A plausible next step for improving the activity of PE nets would be to include a crystallization inhibitor in the matrix that prevents the transformation of the amorphous form of deltamethrin to a less active crystalline form. In sum, whenever designing interventions based on contact insecticides, and whenever a mosquito walks on a crystal, crystallography should be a first step.^47^

**Figure 10:**
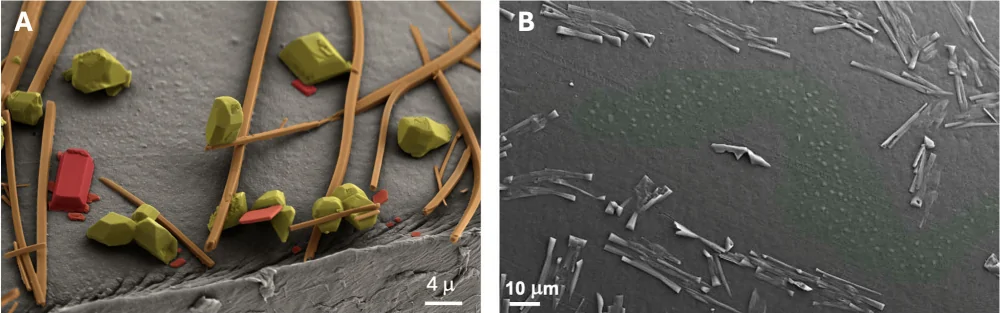
Scanning electron micrographs of PE fiber surfaces. (A) Fiber extruded with 1 wt % ROY, seven days after production showing three distinct morphologies corresponding to three polymorphs (false color). Adapted with permission from ref 46, copyright 2024 American Chemical Society. (B) Fiber extruded with 1 wt % deltamethrin, four days after production showing amorphous deltamethrin droplets diminishing at the expense of Form I crystals. Adapted with permission from ref 35, copyright 2025.
